# Position-Dependent Symptoms of Pneumothorax During Mechanical Ventilation: A Case Report

**DOI:** 10.7759/cureus.50820

**Published:** 2023-12-20

**Authors:** Taku Mayahara, Tomohiro Katayama, Yuki Higashi, Jun Asano, Takashi Sugimoto

**Affiliations:** 1 Emergency Medicine, Kōbe Ekisaikai Hospital, Kobe, JPN; 2 Emergency Medicine, Kobe University Graduate School of Medicine, Kobe, JPN

**Keywords:** lateral decubitus position, body position, pressure controlled ventilation, mechanical ventilation, iatrogenic pneumothorax, pneumothorax

## Abstract

A 54-year-old male with severe hypoxia was transferred to our hospital after choking on a mochi. Chest computed tomography revealed negative pressure pulmonary edema without pneumothorax. Endotracheal intubation was performed, and pressure-controlled ventilation was initiated. Following admission to the intensive care unit, his respiratory condition was stable in both the supine and left decubitus positions. However, every time he was placed in the right decubitus position, the tidal volume decreased by half, and SpO₂ dropped rapidly to 80%, which recovered soon after returning to the supine position. Chest radiography was performed the following day, revealing grade II right pneumothorax, and a chest tube placement stabilized his respiratory status in the right decubitus position. Air leakage ceased within a few hours. Extubation was successful on the fifth hospital day, and the chest tube was removed on the eighth hospital day. To our knowledge, there are no previous reports on position-dependent symptoms of pneumothorax during mechanical ventilation. Clinicians should consider the possibility of pneumothorax on that same side when respiratory deterioration is observed only in one lateral decubitus position during mechanical ventilation.

## Introduction

Timely diagnosis of pneumothorax during mechanical ventilation is essential, as it can quickly progress to tension pneumothorax [1]. Signs of pneumothorax include reduced breath sounds on the affected side, the emergence of new or worsening hypoxia, a decrease in tidal volume during pressure-controlled ventilation (PCV), an increase in airway pressure during volume-controlled ventilation, and symptoms such as tachycardia and hypotension. This case report describes a delayed diagnosis of right pneumothorax during PCV in which hypoxia and reduced tidal volume were observed only in the right lateral decubitus position and not in the supine or left lateral decubitus positions. To the best of our knowledge and search capabilities, there have been no previous reports on position-dependent symptoms of pneumothorax during mechanical ventilation.

## Case presentation

The patient was a 54-year-old male residing in a facility with a height of 170 cm and a weight of 69 kg. He had left hemiplegia and aphasia as sequelae of the cerebral hemorrhage. One afternoon, he developed cyanosis, loss of consciousness, and respiratory arrest due to choking on a mochi. Facility staff contacted the emergency medical services and initiated chest compressions. However, the chest compressions were stopped when the patient appeared to move to brush something off. Upon arrival of the emergency medical team, the patient regained spontaneous respiration with a peripheral capillary hemoglobin oxygen saturation (SpO₂) of 70%. The emergency medical team promptly removed the mochi from the oral cavity, administered oxygen at 10 L/min using a reservoir mask, and transported the patient to our hospital.

**Figure 1:**
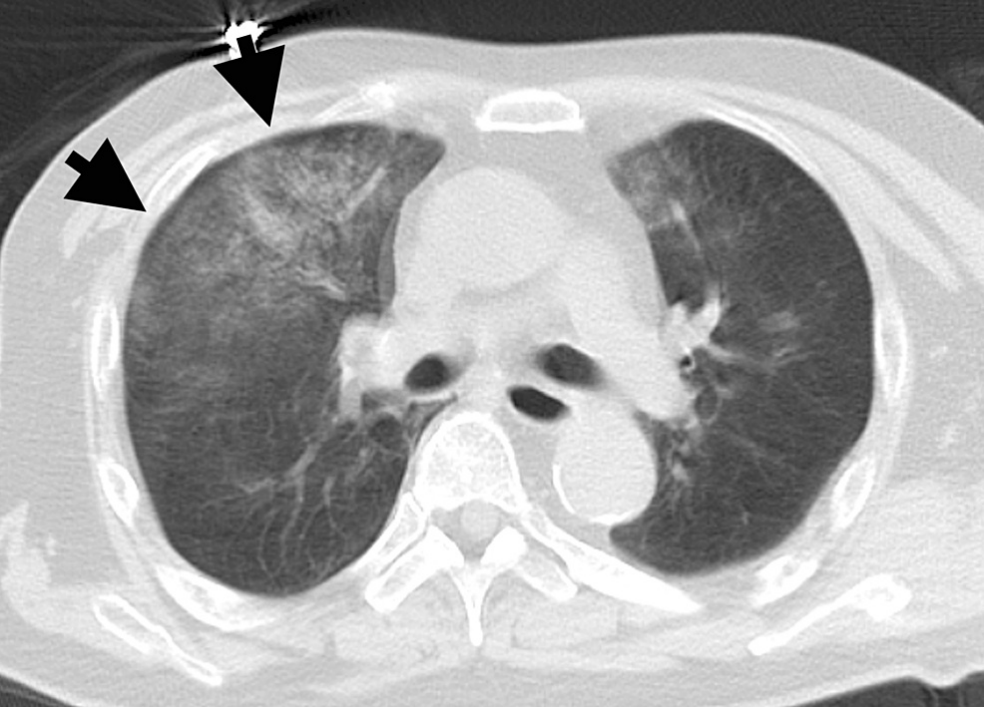
Chest computed tomography upon arrival Infiltration shadow is observed in the upper right lobe, indicates negative pressure pulmonary edema.

Upon arrival at our hospital, his vital signs were as follows: blood pressure, 158/111 mmHg; pulse rate, 88 beats per minute; respiratory rate, 36 breaths per minute; SpO₂ 90% on 10 L/min of oxygen; and Glasgow Coma Scale, E4V1M5. Stridor during inspiration was pronounced, and effortful and paradoxical breathing was observed. Computed tomography (CT) revealed no obstructive object in the airway or pneumothorax but infiltration in the right upper lobe (Figure 1).

**Figure 2:**
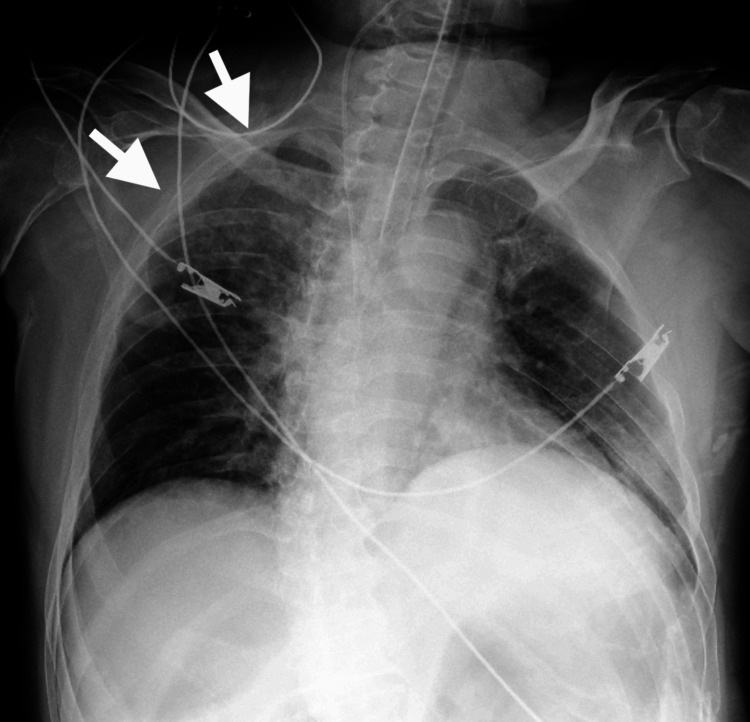
Chest radiography following the placement of the central venous catheter. Chest radiography following the placement of the central venous catheter did not reveal any pneumothorax.

The patient was diagnosed with negative pressure pulmonary edema caused by choking on mochi. The patient was intubated in the emergency department and transferred to the intensive care unit (ICU). Sedation was achieved with propofol and fentanyl. After admission to the ICU, a central venous catheter was placed under ultrasonographic guidance through the right internal jugular vein. The procedure was uneventful, and post-catheterization chest radiography revealed no pneumothorax (Figure 2).

Following the initiation of mechanical ventilation, an initial fraction of inspired oxygen (FiO₂) of 0.8 was necessary to sustain SpO₂ above 90%. However, FiO₂ was decreased to 0.4 within a few hours, resulting in a SpO₂ of 95% or higher. Additional key ventilator parameters included PCV with a peak airway pressure of 15 cmH₂O above the positive end-expiratory pressure (PEEP) to attain a tidal volume of 400 ml, respiratory rate of 14 breaths per minute, and PEEP of 9 cmH₂O.

**Figure 3:**
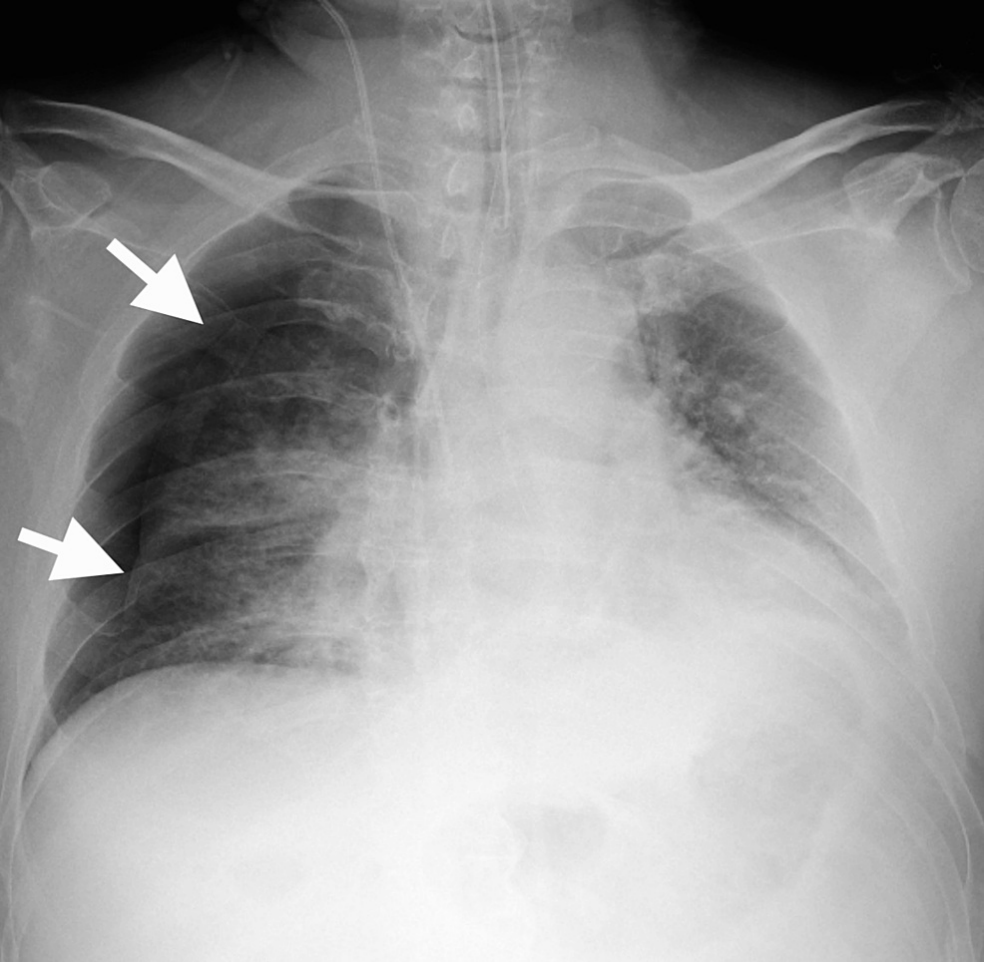
Chest radiography of the following day. Chest radiography performed on the following day revealed second-degree right pneumothorax.

With these ventilator settings, the patient's respiratory condition remained stable in the supine and left lateral decubitus positions. However, when the patient was placed in the right lateral decubitus position for the first time, the tidal volume decreased to less than 200 ml, and SpO₂ dropped to 80% within a few minutes. Blood pressure remained stable without the need for vasopressors. Upon returning to the supine position, the tidal volume and SpO₂ recovered within a few minutes. Auscultation revealed a slight reduction in breath sounds on the right side, which we considered was associated with pulmonary edema predominant in the right upper lobe. Given the stable respiratory condition in the supine and left lateral decubitus positions and the absence of pneumothorax on chest radiography and CT, we excluded the possibility of pneumothorax. The temporary deterioration of the patient's respiratory condition prompted the consideration of atelectasis resulting from retained sputum or unilateral lung intubation due to a position change. Despite attempts to suction the sputum thoroughly and adjust the endotracheal tube position to 1 cm shallower, the situation did not improve. Two more episodes occurred where the tidal volume and SpO₂ decreased shortly after placing the patient in the right lateral decubitus position and quickly recovered upon returning to the supine position. Ultimately, we avoided placing the patient in the right lateral decubitus position until the following morning. The patient's respiratory condition remained stable in the supine and left lateral decubitus positions. The following morning, chest radiography revealed a second-degree right pneumothorax (Figure 3).

After a chest tube placement, the tidal volume immediately increased to exceed 500 ml, necessitating adjustments to the respiratory settings. A decrease in the tidal volume and hypoxia associated with the right lateral decubitus position were entirely resolved. Air leakage ceased within a few hours. Extubation was successful on the fifth hospital day, and the chest tube was removed on the eighth hospital day.

## Discussion

Approximately half of the pneumothoraces in the ICU are reported to be iatrogenic, while the other half are due to barotrauma [1]. In our case, we could not detect pneumothorax on the post-catheterization chest radiography. However, numerous instances of delayed pneumothorax following central venous punctures, including the internal jugular vein, have been reported [2]. The right internal jugular vein puncture may have caused lung injury, and several hours of mechanical ventilation led to the right pneumothorax in our case. Alternatively, it might have been a pneumothorax caused by barotrauma. The prompt cessation of air leakage within a few hours of chest tube placement suggested minimal lung injury.

Two misconceptions contributed to delayed diagnosis in the present case. First, we thought that the symptoms of pneumothorax during mechanical ventilation would progress rapidly, often leading to tension pneumothorax. In the field of blunt chest trauma, there is a condition known as "occult pneumothorax." This condition involves a minor pneumothorax that might escape detection on radiography, requiring diagnosis solely through CT scans. Conventionally, patients with occult pneumothorax who require mechanical ventilation should undergo chest tube placement to prevent tension pneumothorax [1].

In contrast, Mahmood et al. found that among cases of occult pneumothorax requiring mechanical ventilation, 72% were successfully treated without chest tube placement [3]. Our patient's respiratory condition remained stable in the supine and left lateral decubitus positions throughout the treatment, showing no deterioration. However, the patient had a right pneumothorax that was overlooked. It is essential to recognize that even during mechanical ventilation, the symptoms of pneumothorax often remain non-progressive and might resolve conservatively. Although we managed the case with chest tube placement, conservative observation without a chest tube would also have been reasonable.

The second misconception is that the symptoms of pneumothorax during mechanical ventilation would manifest regardless of the patient's position. Our patient experienced significant hypoxia and a reduction in tidal volume when placed in the right lateral decubitus position only. To our knowledge, there is no record of a similar phenomenon in literature. There are two possible explanations for this phenomenon. The first is an exacerbated ventilation-perfusion mismatch. In the lateral decubitus position, gravity increases perfusion in the lower lungs [4]. Because our patient had right pneumothorax and impaired right lung ventilation, increased perfusion of the right lung worsened the ventilation-perfusion mismatch, leading to hypoxia in the right lateral decubitus position. The second pertains to intrathoracic pressure. In the lateral decubitus position, the weights of the abdominal organs place more pressure on the dependent thoracic cavity through the diaphragm [5]. The right pneumothorax of our patient was not severe enough to cause noticeable symptoms in the supine and left lateral decubitus positions. However, in the right lateral decubitus position, the weight of the abdominal organs exerted more significant pressure on the right thoracic cavity, resulting in increased right intrathoracic pressure. This increased right intrathoracic pressure intensified the symptoms of the right pneumothorax, such as hypoxia and reduced tidal volume.

## Conclusions

We experienced a case of delayed diagnosis of right pneumothorax during PCV in which hypoxia and reduced tidal volume were observed only in the right lateral decubitus position and not in the supine or left lateral decubitus positions. If the possibility of pneumothorax had been considered, an earlier diagnosis could have been made using chest radiography or thoracic ultrasonography. Clinicians should consider the possibility of pneumothorax on the same side when respiratory deterioration is observed in only one lateral decubitus position.

## References

[1] Thachuthara-George J (2021). Pneumothorax in patients with respiratory failure in ICU. J Thorac Dis.

[2] Plewa MC, Ledrick D, Sferra JJ (1995). Delayed tension pneumothorax complicating central venous catheterization and positive pressure ventilation. Am J Emerg Med.

[3] Mahmood I, Tawfeek Z, El-Menyar A (2015). Outcome of concurrent occult hemothorax and pneumothorax in trauma patients who required assisted ventilation. Emerg Med Int.

[4] Wieslander B, Ramos JG, Ax M, Petersson J, Ugander M (2019). Supine, prone, right and left gravitational effects on human pulmonary circulation. J Cardiovasc Magn Reson.

[5] Froese AB, Bryan AC (1974). Effects of anesthesia and paralysis on diaphragmatic mechanics in man. Anesthesiology.

